# PAR1-mediated Non-periodical Synchronized Calcium Oscillations in Human Mesangial Cells

**DOI:** 10.1093/function/zqae030

**Published:** 2024-06-10

**Authors:** Mariia Stefanenko, Mykhailo Fedoriuk, Mykola Mamenko, Marharyta Semenikhina, Tamara K Nowling, Joshua H Lipschutz, Oleksandr Maximyuk, Alexander Staruschenko, Oleg Palygin

**Affiliations:** Department of Medicine, Division of Nephrology, Medical University of South Carolina, Charleston, SC 29425, USA; Department of Cellular Membranology, Bogomoletz Institute of Physiology, Kyiv 01024, Ukraine; Department of Medicine, Division of Nephrology, Medical University of South Carolina, Charleston, SC 29425, USA; Department of Physiology, Medical College of Georgia, Augusta University, Augusta, GA 30912, USA; Department of Medicine, Division of Nephrology, Medical University of South Carolina, Charleston, SC 29425, USA; Department of Medicine, Division of Rheumatology & Immunology, Medical University of South Carolina, Charleston, SC 29425, USA; Department of Medicine, Division of Nephrology, Medical University of South Carolina, Charleston, SC 29425, USA; Department of Medicine, Ralph H. Johnson VAMC, Charleston, SC 29401, USA; Department of Cellular Membranology, Bogomoletz Institute of Physiology, Kyiv 01024, Ukraine; Department of Molecular Pharmacology and Physiology, University of South Florida, Tampa, FL 33602, USA; James A. Haley Veterans’ Hospital, Tampa, FL 33612, USA; Department of Medicine, Division of Nephrology, Medical University of South Carolina, Charleston, SC 29425, USA; Department of Regenerative Medicine and Cell Biology, Medical University of South Carolina, Charleston, SC 29425, USA

**Keywords:** thrombin, glomerulus, chronic kidney disease, TRPC channels, SOC entry

## Abstract

Mesangial cells offer structural support to the glomerular tuft and regulate glomerular capillary flow through their contractile capabilities. These cells undergo phenotypic changes, such as proliferation and mesangial expansion, resulting in abnormal glomerular tuft formation and reduced capillary loops. Such adaptation to the changing environment is commonly associated with various glomerular diseases, including diabetic nephropathy and glomerulonephritis. Thrombin-induced mesangial remodeling was found in diabetic patients, and expression of the corresponding protease-activated receptors (PARs) in the renal mesangium was reported. However, the functional PAR-mediated signaling in mesangial cells was not examined. This study investigated protease-activated mechanisms regulating mesangial cell calcium waves that may play an essential role in the mesangial proliferation or constriction of the arteriolar cells. Our results indicate that coagulation proteases such as thrombin induce synchronized oscillations in cytoplasmic Ca^2+^ concentration of mesangial cells. The oscillations required PAR1 G-protein coupled receptors-related activation, but not a PAR4, and were further mediated presumably through store-operated calcium entry and transient receptor potential canonical 3 (TRPC3) channel activity. Understanding thrombin signaling pathways and their relation to mesangial cells, contractile or synthetic (proliferative) phenotype may play a role in the development of chronic kidney disease and requires further investigation.

## Graphical Abstract

**Graphical Abstract:**
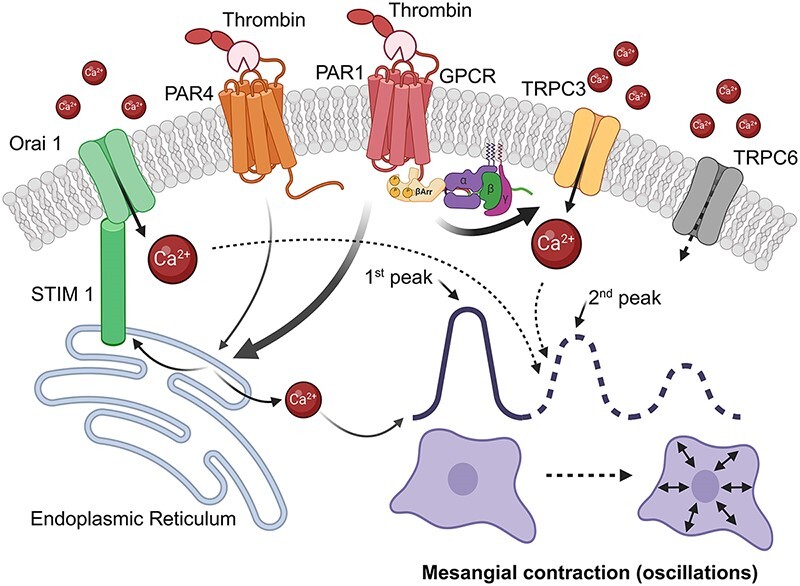

## Introduction

Mesangial cells (MCs) are specialized contractile cells, which abut the glomerular vasculature and basement membrane establishing the central stalk of the glomerulus.^1,2^ MCs generate an extracellular matrix that joins together the basement membrane, glomerular capillaries, and the contractile machinery of MCs.^3^ Aside from structurally supporting the glomerular capillaries, this intertwining network forms a contractile biomechanical unit, allowing for fine-tuning of the intraglomerular blood volume and filtration surface. Indeed, the mechanisms of acute regulation of glomerular blood flow, including the response to changes in the concentration of NaCl in the lumen of the cortically thick ascending limb of Henle or tubuloglomerular feedback mechanism, strongly depend on extraglomerular MC function and the corresponding constriction of intraglomerular afferent arterioles.^4,5^ Moreover, MCs remove molecular aggregates and debris from the basement membrane by phagocytosis, further contributing to maintenance of ultrafiltration. In healthy glomeruli, MCs secrete and respond to numerous signaling molecules, including vasoactive agents, cytokines, and hormones, allowing them to perform a range of physiological functions.^3,6^

MCs have been assiduously studied in relation to the pathogenesis of chronic kidney diseases, such as diabetic nephropathy (DN). Hyperfiltration in the early stages of DN has been associated with functional abnormalities in glomerular mesangium, including decreased contractility and increased surface area.^7^ Excessive proliferation of MCs and deposition of extracellular matrix in the mesangium is a consistent glomerular hallmark of advanced DN.^2^ The resulting mesangial expansion decreases blood flow in glomerular capillaries, and matrix accumulation results in basement membrane thickening and reduction of the glomerular filtration rate.^8^ Ultimately, DN leads to glomerulosclerosis and tubulointerstitial fibrosis characterized by persistent fibrin deposition. Thrombin, a serine proteinase that cleaves fibrinogen into fibrin, is a potent profibrotic factor and a major pathogenetic determinant of DN.^9,10^ In addition to its key role in blood coagulation, thrombin is a potent vasoconstrictor, mitogen, proinflammatory agent, and a powerful activator of several cell types in the glomerulus, including MCs.^11–13^ Thrombin has been shown to stimulate MC proliferation and synthesis of prostaglandins, nitric oxide, endothelin-1, extracellular matrix components, and chemokines.^14,15^ This wide array of functions is achieved by the activation of a G-protein coupled receptor (GPCR), known as protease-activated receptor 1 (PAR1), expressed on the surface of MCs.^16^

PARs are activated by proteolytic cleavage of their extracellular N-terminus.^17^ Thrombin or other serine proteases cleave the receptor at a specific site that acts as a tethered ligand activating the signal transduction through G-proteins. TFLLR-NH_2_ is a synthetic peptide mimicking the tethered ligand, specifically activating PAR1 without thrombin cleavage.^18^ Similarly, other short peptides could be used for activation and allow the study of specific signaling pathways and physiological responses mediated by PAR receptors family.

Activation of PAR1 signaling reportedly contributes to DN. PAR1 expression is upregulated in glomeruli isolated from diabetic db/db mice and streptozocin-induced diabetic rats.^19,20^ Genetic knockout, knockdown, or pharmacological inhibition of PAR1 attenuates DN in streptozotocin-induced diabetic mice and rats.^21,22^ Our recently published data indicate that elevation of serine proteases and PAR1 signaling directly mediate intracellular calcium dynamics in glomerular podocytes.^23^ Moreover, the pathological activation of serine proteases in diabetes further promotes PAR1-TRPC6 (transient receptor potential canonical 6) channel activation, leading to podocyte apoptosis and the development of albuminuria.^24^ It was also reported that PAR1 blockade ameliorates DN and reduces mesangial proliferation in type I diabetic Akita mice with reduced expression of endothelial nitric oxide synthase.^22^ Yet, the specific molecular determinants and the relation of PAR1 signaling to MC function, calcium influx, and corresponding ion channels activation modulating MC contractility are poorly understood. Here, for the first time, we report that the activation of PAR1 mediates intracellular calcium oscillations in primary human renal mesangial cells (HRMCs). Confocal fluorescent microscopy and patch-clamp electrophysiology were combined with pharmacological approaches to dissect the contribution of specific membrane channels to PAR1-mediated calcium signals.

## Materials and Methods

### Cell Culture

Primary HRMCs [male (lot #12445) and female (lot #17554)] were purchased from the ScienCell Research Laboratories (San Diego, CA, USA). In our experiments, we used both male and female cell lines. We did not find significant sex differences in confocal microscopy experiments, and most of the statistical datasets were shown for the male cell line. Cells were cultured in RPMI-1640 medium (Gibco, #11875085), containing Insulin-Transferrin-Selenium (Gibco, #41400045), penicillin streptomycin (Cytiva, #SV30010), and 10% fetal bovine serum (Corning, #35011CV), in a 5% CO_2_ incubator at 37^∘^C. HRMCs in the passages between 4 and 10 were sub-cultured at 90% confluence and used for further experiments. Seeding density was 6 × 10^3^ cells/cm^2^.

### Pharmacological Tools

Thrombin receptor agonist peptide (Tocris, #1185) (water-soluble) at a concentration of 5 μm was used in confocal imaging experiments.^25^ TFLLR-NH_2_ (Tocris, #1464) (water soluble), an established PAR1 selective agonist,^23^ was used in the study to mimic thrombin signaling in HRMCs. Thrombin peptide concentrations used in experiments were slightly higher since, according to the manufacturer, TFLLR-NH_2_ EC_50_ = 1.9 µm and thrombin peptide EC_50_ = 4 µm. AY-NH_2_ (Tocris, #1487) (water soluble), was used as a selective PAR4 receptor agonist peptide.^26^ For PAR1 inhibition, we preincubated cells with 10 µm PAR1 selective inhibitor RWJ 56110 (Tocris Bioscience, #2614) (water soluble).^27^ To inhibit PAR4, cells were preincubated with 50 µm tcY-NH_2_ (Tocris, #1488) (water-soluble).^28^ Cell preincubated for at least an hour with AZ 3451 (100 n m, DMSO soluble), a potent PAR2 antagonist EC_50_=23 n m (MedChemExpress, #HY-112558), to detect PAR2 involvement in thrombin-mediated signaling.^29^ For the inhibition of store-operated calcium entry (SOCE)-related calcium channels (STIM1/Orai1), cells were pretreated with 5 µm of pyrazole compound Pyr6 (DMSO soluble; Sigma-Aldrich, #SML1241).^30^ The Pyr6 concentrations of 15 µm or higher were used to inhibit STIM1/Orai1 and TRPC3 activity.^31^ The selectivity between STIM1/Orai1 and transient receptor potential canonical (TRPC) subfamily channels for the pyrazole compounds was based on the previous reports.^32,33^ The selectivity and concentrations of TRPC 3 and 6 inhibitor GSK 2833503A 20 µm (DMSO/ethanol soluble) (Tocris Bioscience, #6497) and specific TRPC6 inhibitor BI-749327 1 µm (DMSO soluble) (MedChemExpress, #HY-111925, Monmouth Junction, NJ, USA) were used according to previous reports.^30,34^ Purinergic responses were tested with the acute applications of ATP (water soluble) (Thermo Scientific, #R0441).

### Confocal Microscopy

HRMCs were cultured on glass-bottom dishes (Mattek, #P35G-0-14-C, 35 mm dish, No. 0 coverslip, 14 mm glass diameter, uncoated) until reaching 90% confluence, before being utilized for confocal experiments. The cells were loaded with a 5 µm Fluo-8H, AM fluorescent dye (AAT Bioquest, #21090) and incubated at 37°C for 1 h in a CO_2_ incubator. For the RWJ 56110, GSK 2833503A, and tcY-NH_2_ preincubation, the drug or corresponding vehicle were added to the cell media 1 h before the confocal imaging experiments. Before performing the confocal imaging, cells were washed to remove unincorporated dye, and the media was replaced with a 2 m m (or zero) Ca^2+^ extracellular solution (NaCl 145 m m, KCl 4.5 m m, CaCl_2_ 2 (or zero) m m, MgCl_2_ 2 m m, HEPES 10 m m, pH = 7.35). RWJ 56110, GSK 2833503A, tcY-NH_2_, or vehicle were also added to the bath solution after the media was replaced. Pharmacological inhibitors (Pyr6 #SML1241, Sigma-Aldrich; BI-749327 #HY-111925, MedChemExpress, Monmouth Junction, NJ, USA) or vehicle were added to the bath solution 10 min before starting the recording. Confocal imaging was obtained at room temperature using the Leica TCS SP5 (HCX PL APO CS 40×/NA 1.25 Oil) laser scanning confocal microscope system (Leica Microsystems Inc., Deerfield, IL, USA) with dye excitation and emission FITC filter set (488 and 525/25 nm, respectively), and a recording frequency of 0.4 Hz per image. Records were analyzed using ImageJ software (Fiji package).

### Electrophysiology

All electrophysiological experiments were performed at room temperature (22°C-24°C) in the cell-attached voltage-clamp configuration using a MultiClamp 700B Microelectrode Amplifier and Digidata 1550B analog-to-digital signal converter (Molecular Devices, San Jose, CA, USA). The extracellular bath solution consists of 140 m m NaCl, 1.2 m m MgCl_2_, 2.5 m m CaCl_2_, 4.5 m m KCl, 12 m m glucose, 10 m m HEPES; pH 7.4. The patch pipettes (10-12 MΩ) were pulled with a horizontal puller (Sutter *P*-97; Sutter Inst.) and filled with pipette solution containing 80 m m CsCl, 15 m m EGTA, 1 m m MgATP, 1 m m GTP, 4.6 m m CaCl_2_, 10 m m HEPES, 5 m m glucose, 0.1 m m DIDS (Tocris Bioscience, #4523, Minneapolis, MN, USA), 10 n m iberiotoxin (Tocris Bioscience, #1086, Minneapolis, MN, USA), 10 µm nicardipine (Enzo Life Sciences, #ALX-550-273, Farmingdale, NY, USA); pH = 7.4. Recordings were digitized at −60 mV holding potential and a sampling rate of 1 kHz in the Clampex 11.2 software and current activity was analyzed using Clampfit 11.2 software (Molecular Devices, San Jose, CA, USA). Channels activity were determined during the 100 s recording period at baseline and after the acute application of TFLLR-NH_2_ or corresponding pharmacology. The total number of events were used to measure the channels activity within a patch and total current through the clamped membrane were calculated as an integral for the 100 s intervals before and after drug application, as previously reported.^35^

### Glomeruli Volume Dynamics Assay

Sixteen-week Wistar Kyoto male rats were obtained from Charles River and were kept in a light-controlled environment with a 12:12-h light/dark cycle and given free access to water (filtered, RO water) and food (5V75-PicoLab Verified 75 IF, LabDiet, USA). All animal procedures were approved by the Institutional Animal Care and Use Committee at the Medical University of South Carolina in accordance with the Guide for the Care and Use of Laboratory Animals and followed the ARRIVE guidelines.

Experimental procedures were performed as previously described.^36^ Briefly, male Wistar rat kidneys were harvested, decapsulated, and used to isolate glomeruli by differential sieving. Freshly isolated glomeruli were collected and stored on ice in a 15-mL tube with a 5% BSA (Sigma-Aldrich, # A8327)/RPMI-1640 solution with non-permeable 150 kDa TRITC-dextran (1 mg/mL, TdB Labs, #TD150, Uppsala, Sweden). Glomeruli volume changes were measured using fast confocal 3D imaging before and after acute application of 60 µm of Angiotensin II (Bachem, #4006473) or 10 µm of TFLLR-NH_2_. Glomeruli were attached to poly-l-lysine covered glass-bottom dishes and covered with the extracellular solution containing 150 kDa TRITC dextran. Z-stacks with 18 consecutive focal planes (3 µm each) were collected every 1 min, allowing glomeruli volume reconstruction. Output files were imported into Imaris Software (Oxford Bitplane, version 9.6.1), reconstructed in 3D, and processed to calculate glomerular volume.

### Statistics Analysis

Changes in Ca^2+^ fluorescence were calculated for individual cells, with approximately 10 cells per dish analyzed. Each experiment was repeated a minimum of 4 times on the new unexposed cells. For representative Figures, the responses from one single experiment were summarized. The statistical graphs represent an individual cell responses and mean ± SE values. For normalized maximum [Ca^2+^]_i_ amplitudes shown in Figure 6: corresponding vehicle response was taken for 100% and each cell response to drug was normalized to mean vehicle value. Corresponding non-normalized values and statistics for Figure 6 data are shown in Supplemental Figure S1. Data were analyzed with ANOVA, and multiple-comparison adjustments (Tukey post hoc test) were conducted only if the ANOVA *F* value was significant. *P*-values of <0.05 were considered significant. The dose-response curve with variable Hill slope fit was generated by a Nonlinear Curve Fit (DoseResp) module with the corresponding Levenberg-Marquardt algorithm, as recently described.^37^ The generated model was adjusted to improve the adj.*R*-Square ≥0.98. All statistical analyses were performed in OriginPro 2021b software (Microcal Software, Northampton, MA, USA).

## Results

### Basal Activity and Pharmacological Modulation of Ion Channels in HRMCs

**Figure 1.**
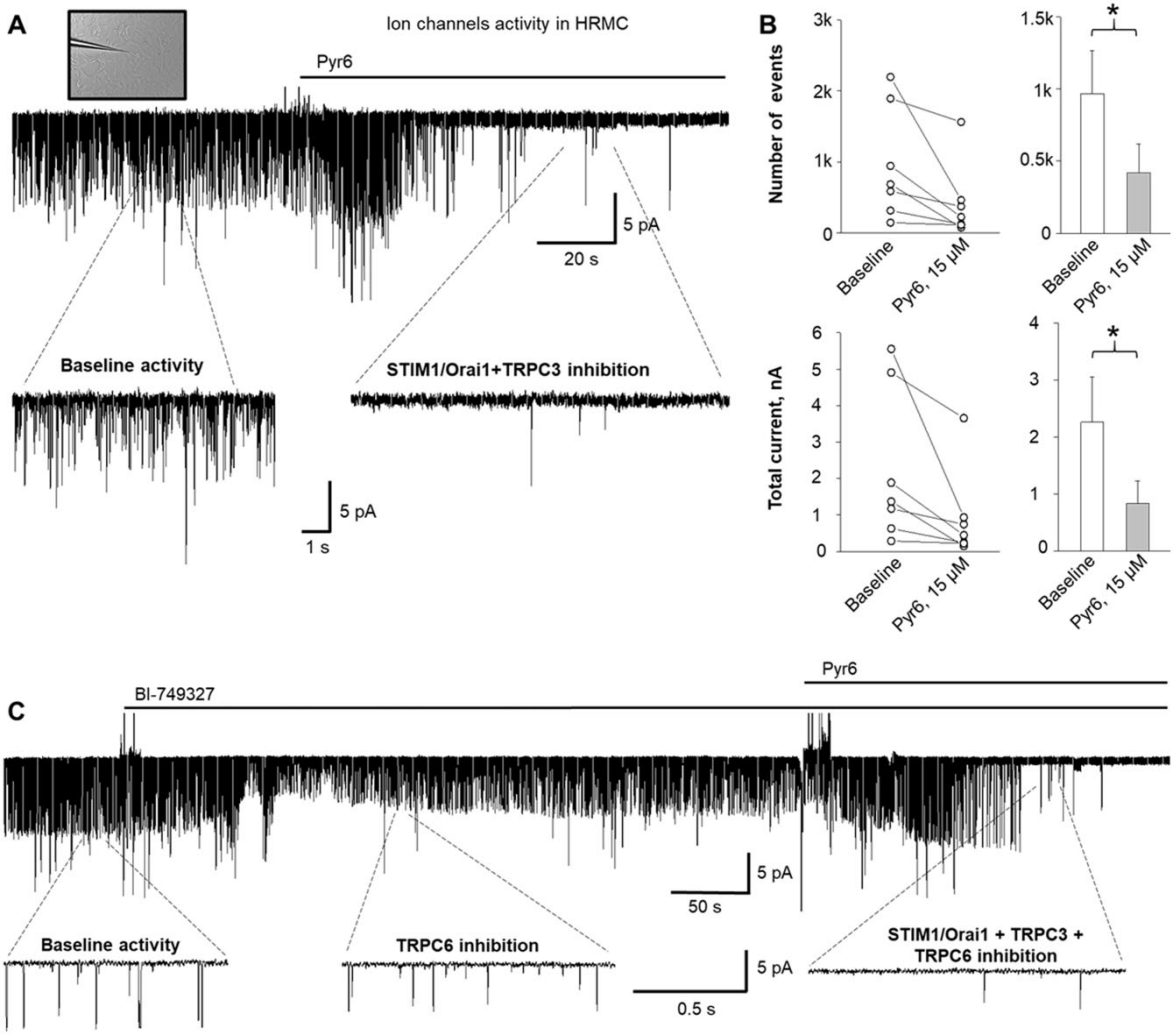
Functional basal activity of STIM1/Orai1, TRPC3, and TRPC6. (A) Representative trace showing inhibition of store-operated calcium entry (SOCE) and TRPC3 baseline activity in human renal mesangial cells (HRMCs) by the acute application of pyrazole derivative Pyr6 (15 µm) (holding potential is −60 mV). (B) The total number of events (top, single channel openings) and total current (bottom, calculated as an integral for the 100 s intervals before and after STIM1/Orai1+TRPC3 inhibition). Shown individual data points (left) and summary graphs (right). Data were analyzed using a one-way RM ANOVA (*P < 0.05). (C) Representative trace showing partial inhibition of baseline activity by TRPC6 channel blocker BI-749327 (1 µm) and strong inhibition of baseline activity by pyrazole derivative Pyr6 (15 µm) in HRMCs (holding potential is −60 mV). Expanded fragments show baseline activity, inhibition of TRPC6 channel after application of BI-749327, and inhibition of STIM1/Orai1+TRPC3 currents after application of Pyr6, respectively.

There are several types of ion channels expressed in MCs.^38^ However, the knowledge about signaling pathways mediated by GPCR PAR and the activation of corresponding ion channels in MCs is limited. To build a strong foundation for our study about the PAR1-mediated calcium influx, we initially explored basal single-channel activity in HRMCs using single-channel electrophysiology. The experiments presented in Figure 1 demonstrated that STIM1/Orai1 complex and TRPC3 channels predominantly contributed to baseline activity, which represent multiple events from different ion channels on the membrane. Inhibition of STIM1/Orai1 and TRPC3 channels with a high concentration of pyrazole derivative Pyr6 (see the “Materials and Methods” section for details) resulted in a significant decrease in basal current activity (Figure 1A and B). In addition, the application of the specific TRPC6 blocker BI-749327 revealed that this channel plays a minor but detectable role in basal activity in HRMCs (Figure 1C).

### PAR1-mediated Calcium Flux in Cultured HRMCs

**Figure 2.**
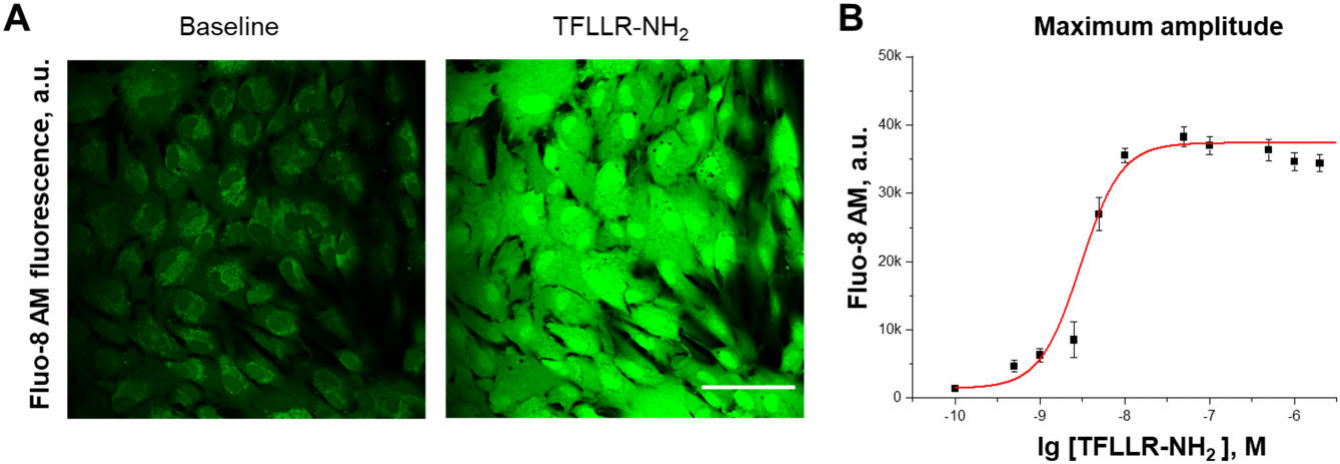
PAR1 response in cultured HRMCs. (A) Representative confocal imaging of intracellular [Ca^2+^]_i_ concentration (Fluo-8H, AM fluorescence) before and after acute application of the PAR1 selective agonist TFLLR-NH_2_ (50 n m). The scale bar is 100 µm. (B) Peak values of PAR1 [Ca^2+^]_i_ release (store release, first peak) fitted by Hill’s equation with half maximal effective concentration (EC_50_) 3.0 ± 0.8 n m.

To explore PAR1-mediated Ca^2+^ flux, we used live cell confocal imaging. HRMCs displayed high sensitivity to a specific PAR1 agonist peptide TFLLR-NH_2_ (Figure 2A). The EC_50_ values were in the nanomolar range of 3.0 ± 0.8 and 6.3 ± 0.2 n m for male- and female-derived cultured cells, respectively. Shown in Figure 2B is the dose-response curve for male HRMCs. The observed sensitivity to a PAR1 agonist peptide was much higher (nanomolar versus tens of micromolar concentration range) in comparison with our reports in other glomerular cells, like podocytes,^24^ or brain cells like astrocytes.^39^ In contrast, Ca^2+^ response to ATP and the activation of corresponding purinergic receptors were in the range of 100 µm (different reports show a 10-100 µm ATP EC_50_ values for MCs^40,41^), which is comparable with previously reported values for podocytes.^42^

### PAR1 Signaling Promotes Intracellular Ca^2+^ Oscillations in HRMCs

**Figure 3.**
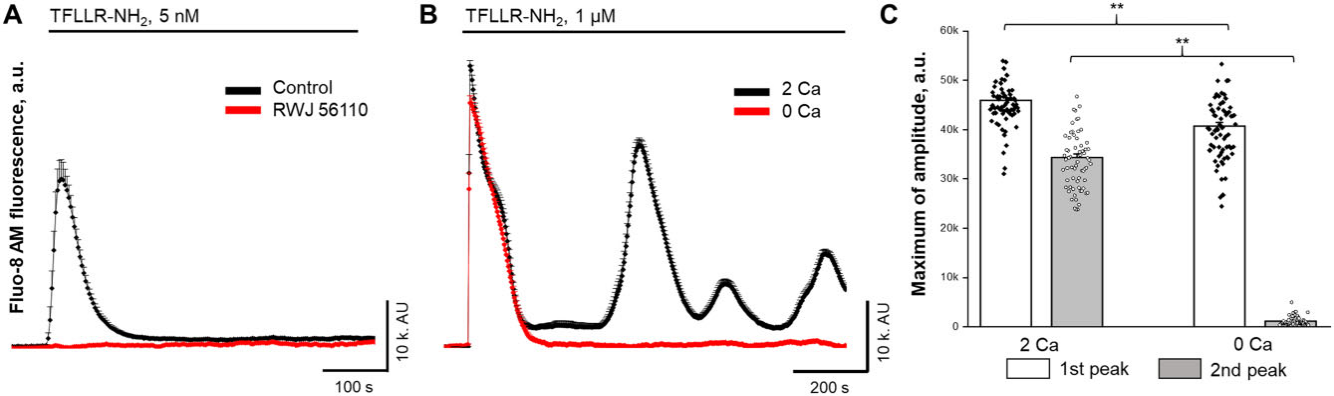
PAR1 signaling promotes intracellular Ca^2+^ [Ca^2+^]_i_ oscillations in human renal mesangial cells. (A) Confocal imaging experiment (Fluo-8H, AM fluorescence) show a small dose (5 n m) of PAR1 (TFLLR-NH_2_) agonist peptide promotes fast [Ca^2+^]_i_ response (black line/single-peak line). The preincubation of cells with the specific PAR1 inhibitor (RWJ 56110, 10 µm) eliminated PAR1-mediated [Ca^2+^]_i_ release (flat line). (B) Confocal imaging experiment shows a saturated concentration of TFLLR-NH_2_ (1 µm, see dose response in Figure 2) promotes synchronized damped Ca^2+^ oscillations (black line/multiple-peak line). The oscillations disappeared in a zero Ca^2+^ extracellular solution (single-peak line). (C) Summary for confocal experiments shown mean (bars) and individual cell (data points) of maximum [Ca^2+^]_i_ amplitudes for first (store release, first peak, white bar/closed circle) and second (extracellular influx, second peak, gray bar/open circle) peaks in response to TFLLR-NH_2_ (1 µm). One way ANOVA, **P < 0.001 between different Ca^2+^ extracellular solution concentrations (2 or 0 m m of Ca^2+^).

**Figure 4.**
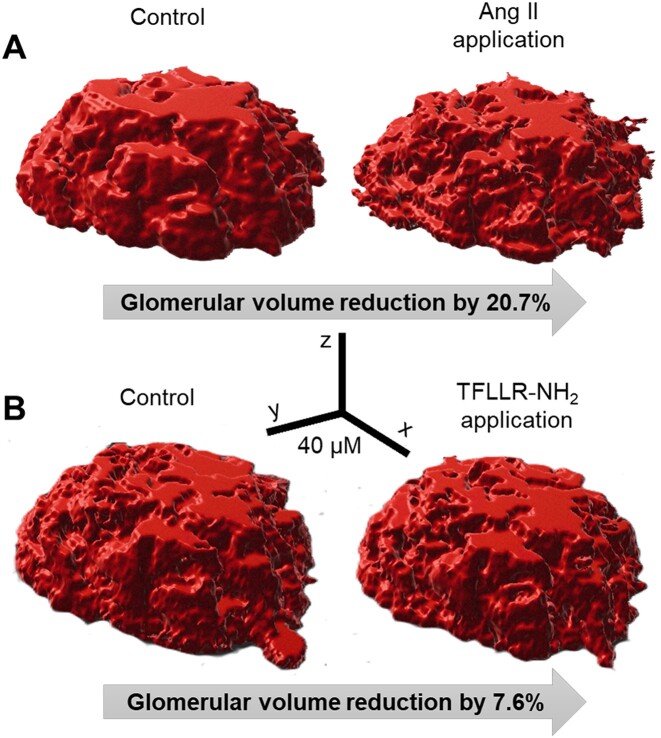
The contraction of the glomerular mesangial matrix in response to Ang II and PAR1 agonist acute applications. (A) Fast confocal 3D imaging shows changes in glomerular volume in response to acute application of Ang II (60 µm). (B) Fast confocal 3D imaging shows changes in glomerular volume in response to acute application of PAR1 agonist peptide TFLLR-NH_2_ (10 µm). The volume changes were calculated using the Imaris Image Analysis Software package.

We further decided to explore PAR-mediated calcium oscillations in MCs, given the smooth muscle-like nature of these cells. It is known that smooth muscle cells’ functional contractility is mediated by periodic pulses of cytosolic Ca^2+^ trigger oscillations, which may be responsible for contraction and membrane depolarization that couples the individual oscillators together, mediating the synchronization.^43^ First, the acute application of TFLLR-NH_2_ in the nanomolar range resulted in a single calcium peak, which could be blocked by preincubation with a specific PAR1 signaling antagonist, RWJ 56110 (Figure 3A). However, when we increased the TFLLR-NH_2_ concentration up to saturation levels of 1 µm and achieved maximum PAR1 signaling activation (see Figure 2B), the initial increase in cytosolic Ca^2+^ levels was followed by synchronized, damped Ca^2+^ oscillations (repeated calcium peaks appeared after the initial one) with a lag of 6.74 ± 0.84 minutes between first and second peaks (Figure 3B and Supplemental Video S1). Notably, the oscillation pattern was absent in extracellular zero calcium solutions, suggesting a crucial role for ionotropic Ca^2+^ entry and resident ion channel activation (Figure 3B and C). To make a more direct connection between the Ca^2+^ oscillations and presumed cellular contractions we performed glomerular volume changes assay using freshly isolated Wistar rat glomeruli. The acute application of Ang II result in strong contraction of the mesangium matrix and reduce glomerular volume up to 20.7% (Figure 4A). The application of PAR1 activating peptide TFLLR-NH_2_ resulted in the volume reduction up to 7.6% (Figure 4B).

### Intracellular Ca^2+^ Oscillations in Response to Thrombin and Role of PAR1

**Figure 5.**
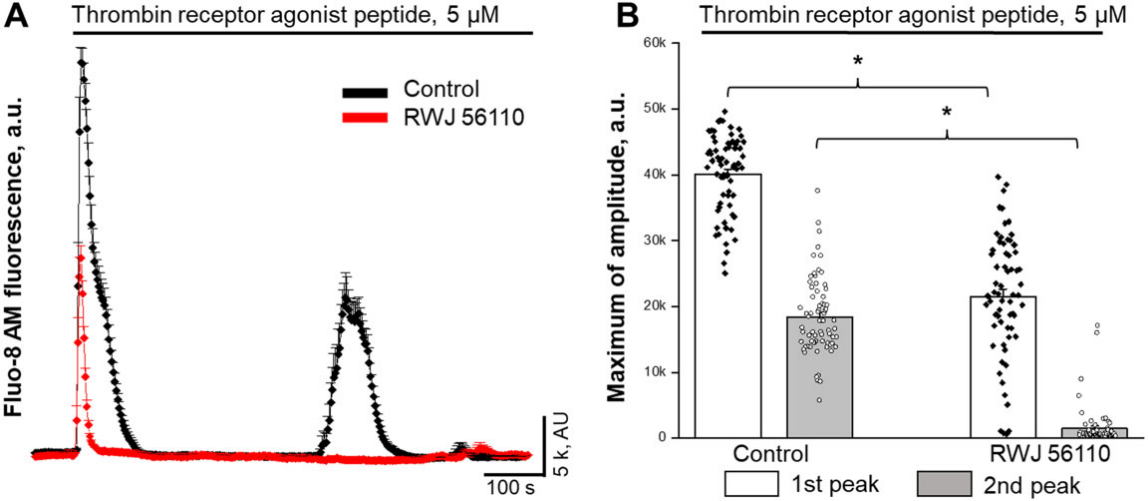
The activation of protease-activated receptor 1 (PAR1) is required for thrombin-mediated intracellular Ca^2+^ [Ca^2+^]_i_ oscillations in human renal mesangial cells. (A) Confocal imaging experiment (Fluo-8H, AM fluorescence) shows [Ca^2+^]_i_ oscillations in response to application of thrombin receptor agonist peptide (5 µm) (black line). The preincubation of cells with the specific PAR1 inhibitor (RWJ 56110, 10 µm) significantly inhibit 1^st^ peak (store release, white bar) and eliminate 2^nd^ peak (extracellular influx, gray bar) of [Ca^2+^]_i_ response to thrombin in HRMCs (red line). (B) Summary for confocal experiments shown mean (bars) and individual cell (data points) of maximum [Ca^2+^]_i_ amplitudes for first (store release, 1^st^ peak, white bar) and second (extracellular influx, 2^nd^ peak, gray bar) peaks in response to thrombin receptor agonist peptide (5 µm) with or without the presence of PAR1 antagonist RWJ 56110. One way ANOVA, ** p<0.001 between control and RWJ 56110 treated groups.

**Figure 6.**
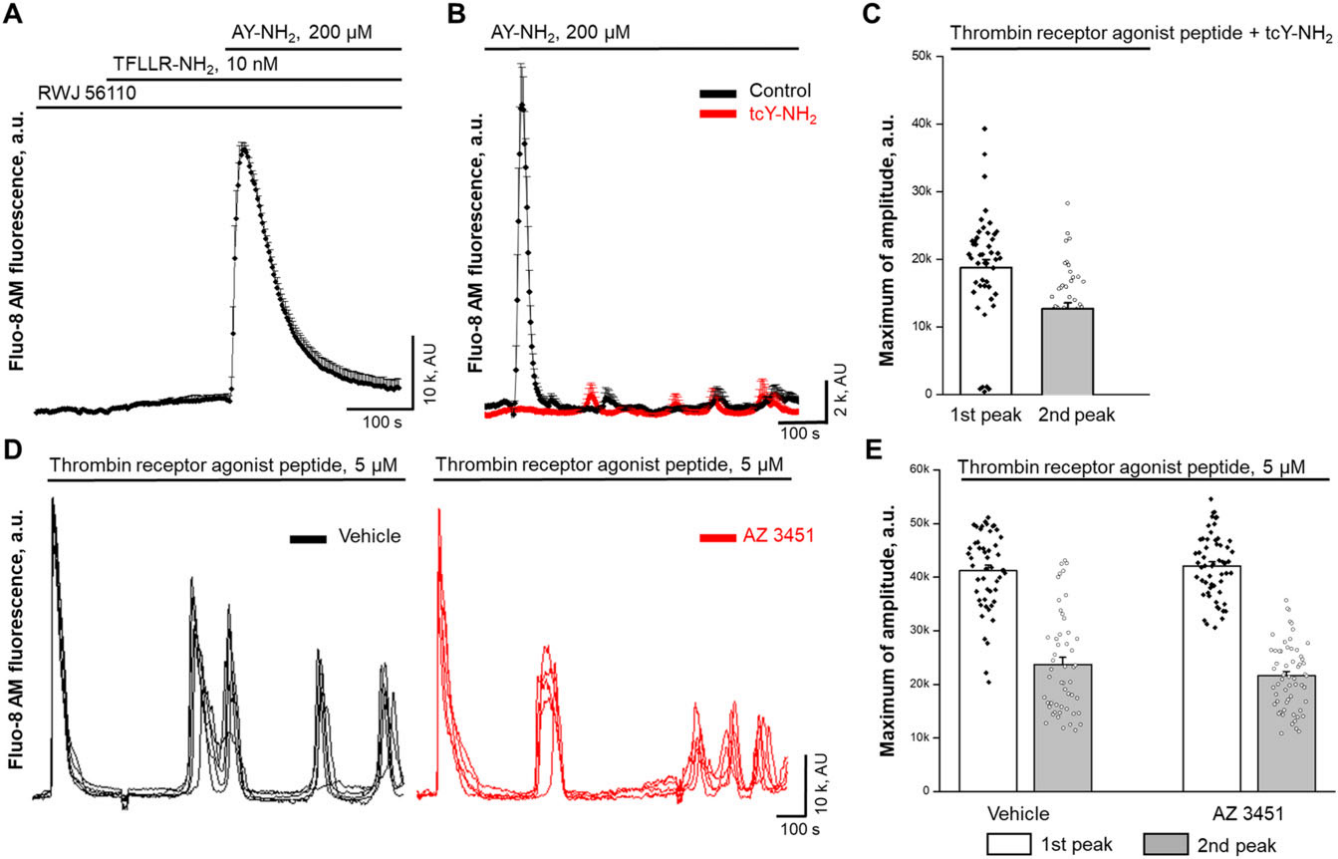
The activation of protease-activated receptor 4 or 2 (PAR4 or PAR2) is not required for thrombin-mediated intracellular Ca^2+^ [Ca^2+^]_i_ oscillations in human renal mesangial cells. (A) Confocal imaging experiment (Fluo-8H, AM fluorescence) shows the presence of PAR4-mediated [Ca^2+^]_i_ in HMRCs. Cells preincubated with the specific PAR1 inhibitor (RWJ 56110, 10 µm) were not responsive to PAR1 agonist peptide TFLLR-NH_2_ (10 n m), but produced robust transient in response to PAR4 agonist peptide AY-NH_2_ (200 µm). (B) Confocal imaging experiment (Fluo-8H, AM fluorescence) shows the absence of synchronized [Ca^2+^]_i_ oscillations in response to PAR4 agonist peptide AY-NH_2_ (200 µm) (black line). The preincubation of cells with the specific PAR4 inhibitor (tcY-NH_2_, 50 µm) inhibits the response entirely. Note the presence of non-synchronized spontaneous [Ca^2+^]_i_ sparks in individual cells in both records. (C) Summary for confocal experiments shown mean (bars) and individual cell (data points) of maximum [Ca^2+^]_i_ amplitudes in response to thrombin receptor agonist peptide (5 µm) with the presence of the specific PAR4 inhibitor (tcY-NH2, 50 µm). (D) The example of synchronized non-periodical oscillations in individual cells after acute application of thrombin receptor agonist peptide in the presence of vehicle (DMSO, black line) or PAR2 agonist (AZ 3451, 100 n m, red line). (E) Summary for confocal experiments shown in D. Mean (bars) and individual cell (data points) of maximum [Ca^2+^]_i_ amplitudes in response to thrombin receptor agonist peptide (5 µm) with the presence of the specific PAR2 inhibitor (AZ 3451, 100 n m).

Beyond the coagulation functions, thrombin is a crucial activator of PARs signaling, which primarily activates PAR1 and PAR4 on platelets and endothelial cells, initiating a cascade of intracellular signaling pathways. To test if thrombin promotes cytosolic Ca^2+^ oscillations, we performed confocal imaging experiments using acute applications of thrombin peptide. As shown in Figure 5A, the application of thrombin promotes synchronized Ca^2+^ oscillations, similar to PAR1 agonist peptide TFLLR-NH_2_ (see Supplemental Video S2). Interestingly, preincubation of cells with specific PAR1 antagonist RWJ 56110 significantly inhibits the first peak (store release, see Figure 3B) and eliminates the second peak, responsible for the extracellular influx and oscillations (Figure 5A and B, and Supplemental Video S3). We perform the following experiments to explore further if the PAR4 signaling cascade may be involved in MC signaling. HMRCs were preincubated in RWJ 56110 to block PAR1 response on the acute application of TFLLR-NH_2_ peptide. The following application of PAR4 activating peptide AY-NH_2_ promotes robust intracellular Ca^2+^ release (Figure 6A), confirming the functional presence of PAR4 in MCs. In the prolonged recording, the same concentrations of PAR4 activating peptide did not promote synchronized Ca^2+^ oscillations and can be efficiently blocked by the application of PAR4 antagonist tcY-NH_2_ (Figure 6B). Moreover, the applications of thrombin in the presence of tcY-NH_2_ and corresponding PAR4 blockade successfully produce Ca^2+^ oscillations, as evidenced by statistical data showing the presence of a second peak in Figure 6C.

In addition, we performed a series of experiments to test the possible involvement of PAR2 in the described Ca^2+^ oscillation behavior in response to thrombin. As shown in Figure 6D, thrombin-mediated oscillations in individual cells are not affected by the application of the PAR2 agonist AZ 3451. The statistical analysis (Figure 6E) indicates that preincubation with AZ 3451 also does not affect the first (store release) peak.

### Pharmacological Inhibition of PAR1-mediated Oscillations

**Figure 7.**
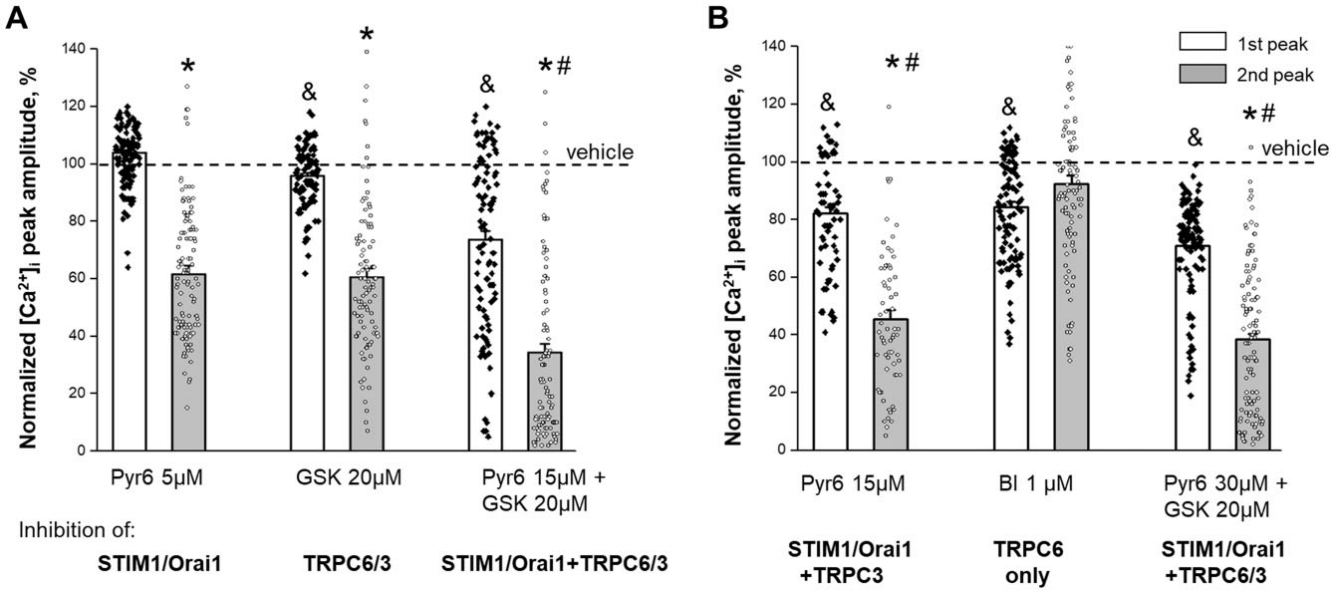
Pharmacological inhibition of PAR1-mediated oscillations in human renal mesangial cells. (A) Summary for confocal experiments shown mean (bars) and individual cell (data points) of the normalized maximum [Ca^2+^]_i_ amplitudes during the pharmacological blockade of SOCE (STIM1/Orai1) entry (Pyr6 5 µm), ionotropic TRPC6 and TRPC3 channels (GSK 2833503A 20 µm) influx, and both STIM1/Orai1 + TRPC6/3 (Pyr6 15 µm + GSK 20 µm). (B) Summary for confocal experiments shown mean (bars) and individual cell (data points) of the normalized maximum [Ca^2+^]_i_ amplitudes during the pharmacological blockade of STIM1/Orai1 + TRPC3 (Pyr6 15 µm), ionotropic TRPC6 only (BI-749327, 1 µm), and STIM1/Orai1 + TRPC6/3 (Pyr6 30 µm + GSK 20 µm). Graphs show the [Ca^2+^]_i_ maximum amplitude normalized to mean vehicle response (see Supplemental Figure S1 for not normalized to vehicle values). White/closed circles and gray/open circles bars/data points indicate store release (first peak) and extracellular influx (second peak). Two-way ANOVA with Dunnett’s post hoc test, vehicle versus drugs application, ^&^*P* < 0.01 (for first peak), *P < 0.0001 (for second peak). Two-way ANOVA with Tukey post hoc test, Pyr6 5 µm or GSK alone versus Pyr6 15 µm or drug combination, ^#^*P* < 0.0001 (for second peak).

PARs are G protein-coupled receptors and may adopt distinct active conformations and signal to diverse effectors in many cells.^44^ According to the data above, PAR1 signaling in HRMCs consists of initial intracellular store release and synchronized oscillations mediated by plasma membrane ion channels. To dissect the contribution of the store-operated (Orai1) and second messenger-operated (TRPC) plasma membrane channels to the calcium oscillations, we performed confocal imaging experiments in the presence of specific pharmacological blockers (see the  “Materials and Methods” section for detailed information about drugs and vehicle). We measured the changes in the intracellular Ca^2+^ amplitude of the first response (depo release) and the amplitude of the second peak (representing ionotropic influx) after applying 1 µm TFLLR-NH_2_ in the presence of a vehicle or corresponding drugs (Supplemental Figure S1). Pyr6, which inhibits the store-operated calcium (SOCs) channels at the low (5 µm) concentration, significantly attenuated the oscillation amplitudes (represented as the maximum amplitude of the second calcium peak; Figure 7A). Similarly, the TRPC3/6 inhibitor (GSK 2833503A), reduced the oscillation amplitude up to 40% (two-way ANOVA, *P < 0.0001 compared to vehicle). The simultaneous application of both drugs resulted in an additive effect with a total of 65% blockade of the second peak amplitude (two-way ANOVA, *P < 0.0001 compared to vehicle) (Figure 7A). Further experiments shown in Figure 7B include high Pyr6 concentration (over 15 µm) with or without a TRPC3/6 inhibitor (GSK 2833503A) to simultaneously block STIM1/Orai1 + TRPC3 channels and blockade TRPC6 with a selective inhibitor BI-749327. Note, that preincubation with high Pyr6 concentration (Figure 7B) also cause significant inhibition of first response, and then may reflect on overall deficit in intracellular Ca^2+^ pool, which later may significantly inhibit oscillations amplitudes. These studies indicate that oscillation is presumably mediated by the activation of STIM1/Orai1 and TRPC3 channels and does not depend on TRPC6 activity.

### Single Channel Activity in Response to PAR1 Signaling Activation in HRMCs

**Figure 8.**
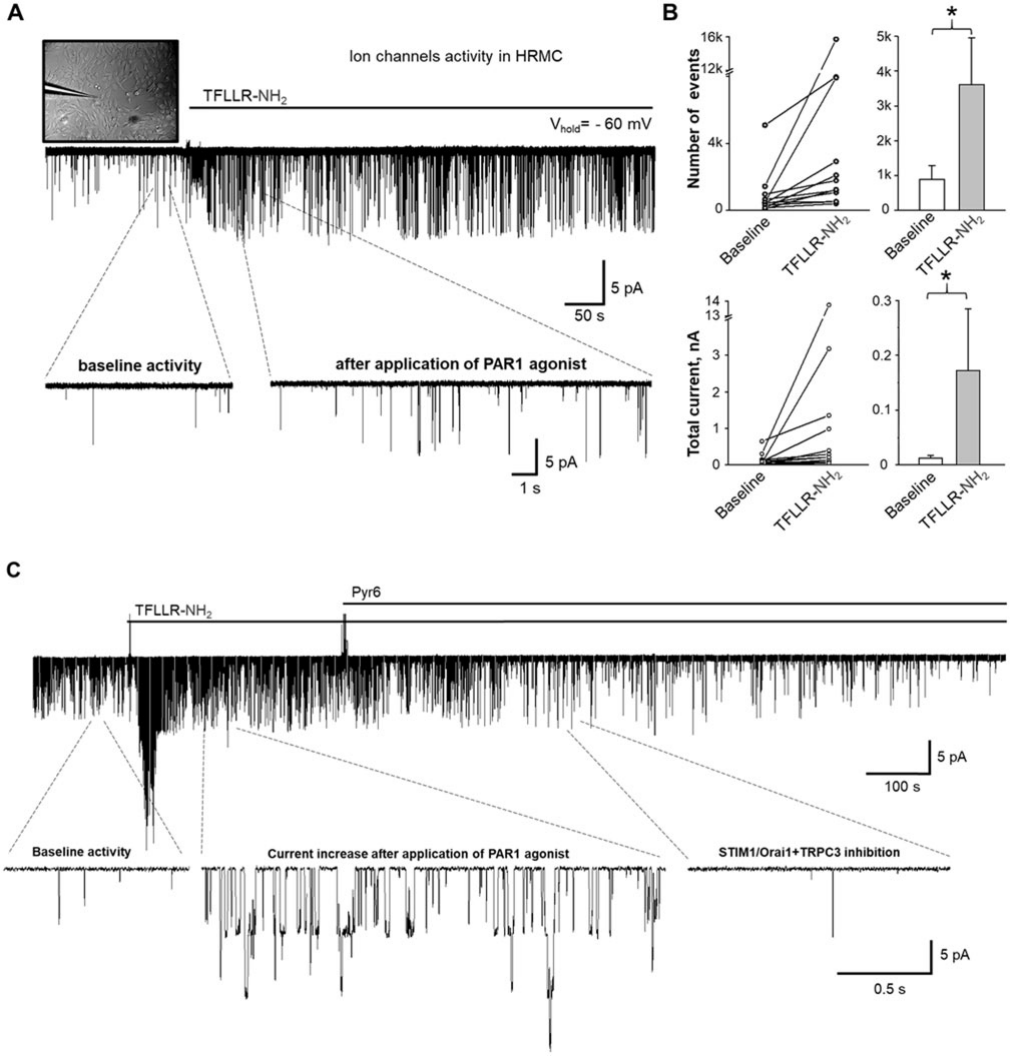
G-protein-coupled receptors PAR1 signaling activate TRPC3 and STIM1/Orai1 channels in human renal mesangial cells (HRMCs). (A) Representative electrophysiological recording of channels activity in HRMCs before and after application of PAR1 agonist peptide TFLLR-NH_2_ (1 µm). The top left corner shows a photomicrograph of the electrophysiological experiment. The single-channel trace insets show expanded recording intervals. (B) The total number of events (top, single channel openings) and total current (bottom, calculated as an integral for the 100 s intervals before and after PAR1 activation) changes in response to TFLLR- NH_2_ (1 µm) application. Shown individual data points (left) and summary graphs (right). One-way RM-ANOVA, *P < 0.05. (C) Representative trace showing the activation of PAR1 by the specific agonist peptide TFLLR-NH_2_ (1 µm) and inhibition of PAR1-mediated STIM1/Orai1+TRPC3 channels activity by pyrazole derivative Pyr6 (15 µm). Expanded fragments show baseline activity, increased current activity after application of TFLLR-NH_2_, and inhibition of currents after application of Pyr6, respectively. All traces were recorded at −60 mV holding potential.

We used patch clamp electrophysiology to confirm our findings to reveal ion channel activation in response to acute PAR1 agonist application. The addition of saturated concentrations of TFLLR-NH_2_ (1 µm) into the bath solution promoted a rapid increase in a number of events and overall current density (Figure 8A and B), suggesting the presence of ion channel-mediated calcium influx which was observed in the confocal microscopy experiments in Figure 3B. Similarly, PAR1-mediated ion channels activity could be significantly reduced by application of a high concentration of Pyr6 (15 µm) and blockade of STIM1/Orai1+TRPC3 channels (Figure 8C).

## Discussion

The seminal studies from the 90’s highlighted that thrombin is a potent regulator of MC function, influencing not only the contractile behavior of these cells but also their role in matrix production and overall renal physiology.^14,45^ Thrombin’s actions are multifaceted, impacting cellular processes ranging from contraction to biochemical mediator synthesis, which are essential for the normal and pathological states of the kidney’s filtering mechanism. Importantly, the high sensitivity of MCs to thrombin observed in our studies with specific PAR1 agonist peptide (Figure 2) was supported by the similar thrombin applications, where significant Ca^2+^ response was detected at concentrations around 0.1 U/mL (approximately 1 n m).^14,46^ As mentioned above, podocytes express PARs but require much higher concentrations for the intracellular Ca^2+^ transient. The other cells with known functional expression of PARs are platelets, endothelial, vascular smooth muscle, monocytes and macrophages, and neuronal cells could be divided into three groups for their sensitivity to thrombin:

Some cell types, such as platelets, are highly sensitive to thrombin. Even low concentrations (0.1 to 1 U/mL) are sufficient to induce Ca²⁺ mobilization in these cells, leading to platelet secretion and aggregation.^47^

Moderate sensitivity. Concentrations around 0.5 to 2 U/mL are often required to activate endothelial and vascular smooth muscle cells.^48^ Endothelial cells utilize thrombin to regulate barrier function and inflammatory responses through Ca²⁺ signaling.^49^ Vascular smooth muscle cells primarily use PAR signaling for contraction and proliferation, which is also an important property for mesangial cells.

Other cell types, such as monocytes and neuronal cells, demonstrate a lower sensitivity to thrombin. These cells generally require higher thrombin concentrations (1-5 U/mL) to activate intracellular Ca²⁺ signaling. This activation plays a role in monocyte adhesion and migration, which is crucial for inflammatory responses.^50^ In the nervous system, thrombin can influence neuron and glial function, as we have also described in our previous studies,^32^ impacting cell survival and inflammatory processes.^51^

Our data suggest that MCs appear to have high sensitivity, similar to platelets, to promote initial Ca²⁺ response but require higher close to moderate range concentrations to reveal Ca²⁺ oscillations.

**Figure 9.**
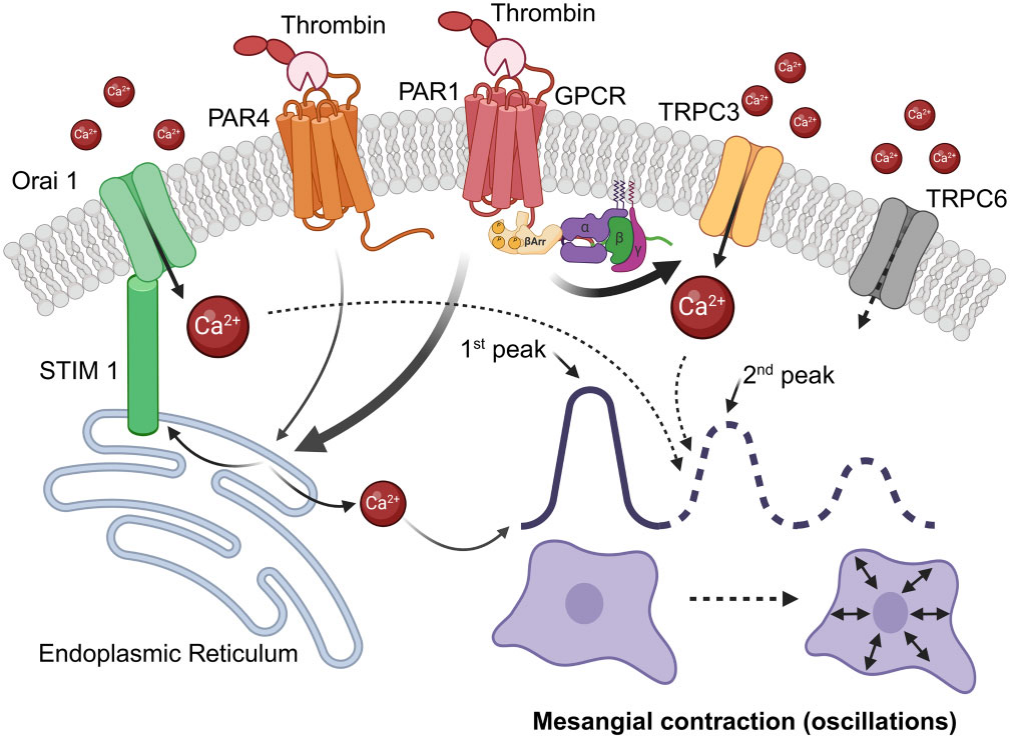
Saturated PAR1 agonist concentrations, in addition to SR/ER calcium release, activate ionotropic calcium influx from STIM1/Orai1 and TRPC3 channels, mediating intracellular calcium oscillations.

Intracellular calcium is an essential second messenger regulating multiple aspects of MC function, modulating responses to vasoactive hormones, polypeptide growth factors, and cytokines.^2,38,52^ Importantly, changes in intracellular calcium concentration are the key requirement for cell contractility. Thus, calcium flux and corresponding MC contraction can directly modulate glomerular blood flow and renal hemodynamics.^7^ Thrombin and PARs are known to mediate intracellular calcium signaling and activate a variety of intracellular cascades.^47,53^ An activation of the PAR1 receptor can stimulate G_q_ protein and trigger subsequent IP_3_-dependent calcium release from the sarco/endoplasmic reticulum (SR/ER).^54^ Calcium mobilization depletes the intracellular stores and triggers the opening of calcium channels on the plasma membrane. The resulting calcium influx from the extracellular compartment allows replenishment of intracellular stores and is termed SOCE. Numerous findings support the presence of SOCE in MCs,^52^ where it can be mediated by a highly calcium-selective ORAI1 channel or nonselective transient receptor potential canonical channels, such as TRPC1, 3, 4, and 6.^38^ However, TRPC channels can be activated independently as receptor-operated calcium channels by various ligands, including via the PAR1 pathway.^24,55^ Our data suggest that PAR1 activation in MCs triggers calcium release from the SR/ER at nanomolar concentrations. Moreover, saturated PAR1 agonist concentrations, in addition to SR/ER calcium release, activate ionotropic calcium influx from STIM1/Orai1 and TRPC3 channels and mediate intracellular calcium oscillations (Figure 9). The observed spontaneous cellular oscillations are synchronized between cells in the monolayer and may represent a typical smooth muscle contractility phenotype.^43^ MC oscillations can be characterized by a quick spike in Ca²⁺ followed by a more extended phase of oscillations, which takes minutes (our recordings usually take up to 20 min; see supplemental video for more information). It is important to note that MC may possess properties similar to vascular smooth muscle cells, and cytosolic Ca^2+^ oscillations may lead to cell contraction or proliferation. Moreover, under pathophysiological conditions, this type of cells may develop either contractile or synthetic (proliferative) phenotype.^56^ The questions are essential for understanding the physiology and pathophysiology of mesangial cells since both mesangial matrix contractility and proliferation are the basis of most glomerular diseases.

Our data suggest the selective role of PAR1, and not PAR4 or PAR2, in triggering cytosolic Ca²⁺ oscillations in MCs. While both receptors engage distinct GPCR mechanisms, PAR1 mediates its effects through interactions with Gαq, Gαi, and Gα12/13 proteins, facilitating rapid and versatile cellular responses. Conversely, PAR4, which primarily associates with Gαq and Gα12/13, exhibits a slower activation response but maintains signaling over a longer period.^57^ This prolonged activation is crucial for sustained thrombin signaling in the context of chronic disease progression and role of PAR4 required further detailed investigation. Our study contributes to the broader understanding of GPCR-mediated calcium signaling oscillations, illustrating its complexity and diversity across different cell types, including MCs.^58^

Mounting evidence links SOCE in MCs to extracellular matrix protein synthesis and deposition.^59–62^ Changes in SOCE parallel mesangial expansion and the fibrotic glomerular phenotype in DN.^52,61^ Moreover, PAR1 overactivation and high activity of serine proteases have recently been linked to glomerular pathologies like FSGS and DN.^24,55^ Interestingly, the above-mentioned studies emphasize the key role of TRPC6 channels in PAR1-mediated podocyte and glomerular damage. In contrast, MCs mediate contractility through the STIM1/Orai1 and TRPC3 channels, where the expression of TRPC6 plays a minor role and does not significantly contribute to the observed phenomenon. Our data indicate high sensitivity of MCs to the PAR-1 activating peptide, which may contribute to both physiological and pathophysiological functions. For instance, high serine proteases activity may induce frequent cytosolic oscillations and changes in membrane potential in MCs, which can lead to hyperfiltration and glomerular blood flow disturbance or mesangial matrix accumulation and consecutive aberrant mesangial cell proliferation leading to glomerulosclerosis. Our study provides direct evidence of the possible role of PAR1 receptors in glomerular and MC pathology and raises the question about the potential use of PAR1 as a therapeutical target in glomerular diseases. Therefore, further investigation of this pathway, including research using various animal models, is required.

## Supplementary Material

zqae030_Supplemental_Files

## Data Availability

The data underlying this article are available in the article and from the corresponding author upon reasonable request. Supplemental Video S1 PAR-1 mediated synchronized non-periodical [Ca^2+^]_i_ (Fluo-8H, AM) oscillations in HRMCs in response to application of specific short peptide activator TFLLR-NH_2_  https://doi.org/10.6084/m9.figshare.25748973.v1; Supplemental Video S2 PAR-1 mediated synchronized non-periodical [Ca^2+^]_i_ (Fluo-8H, AM) oscillations in HRMCs in response to thrombin peptide application https://doi.org/10.6084/m9.figshare.25748976.v1; Supplemental Video S3 mediated the absence of synchronized non-periodical [Ca^2+^]_i_ (Fluo-8H, AM) oscillations in HRMCs in response to thrombin application in the presence of specific PAR1 antagonist RWJ 56110 https://doi.org/10.6084/m9.figshare.25748979.v1 are also available at Figshare.
