# Supplementary figures and images for: PAR1-mediated Non-periodical Synchronized Calcium Oscillations in Human Mesangial Cells
Source: Function (Oxf). 2024 Jun 10;5(5):zqae030. doi: 10.1093/function/zqae030 (PMC11384906; doi:10.1093/function/zqae030)

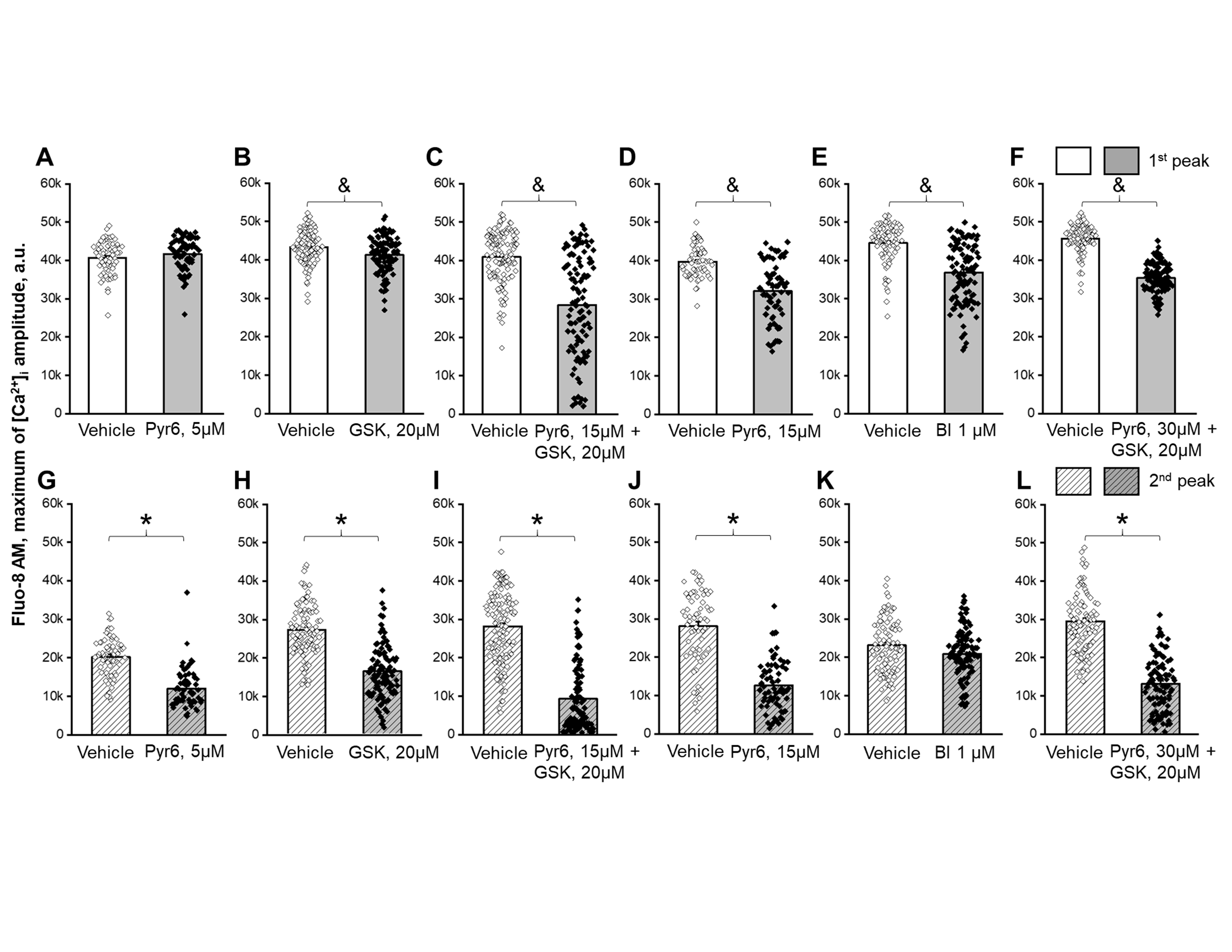

Supplement: zqae030_Supplemental_Files [file zqae030_supplemental_files.zip › Supplemental Figure S1.tif]
